# Daily Stress from Behavioral Symptoms of Dementia Among Hispanic and Latino Caregivers: The Role of Social Isolation and Functional Limitations

**DOI:** 10.1002/alz70858_097847

**Published:** 2025-12-24

**Authors:** Frank Puga, Natashia Bibriescas, Loreli Alvarez, Stephanie DiFiglia, Andres Azuero, Michael G Crowe

**Affiliations:** ^1^ University of Alabama at Birmingham, Birmingham, AL, USA; ^2^ MJHS Institute for Innovation in Palliative Care, New York, NY, USA

## Abstract

**Background:**

Hispanic and Latino (H&L) populations are disproportionately impacted by Alzheimer's disease and related dementias (ADRD) and rely heavily on informal care from family and friends, who often face high caregiving burden and stress. Behavioral symptoms of dementia (BSD) are a significant source of caregiver stress, yet little is known about daily variations in BSD‐related stress or the factors that increase or reduce it. This study examined how caregiver characteristics, care recipient functional limitations, and caregiver‐perceived social isolation influence BSD‐related stress among H&L caregivers.

**Methods:**

H&L caregivers (*n* = 168) completed daily diary surveys over 21 consecutive days, with a total of 3,054 recorded observations. These surveys measured daily BSD‐related stress overall and by individual BSD. The most stressful BSDs were identified using mean stress levels and deviations from baseline stress. Multilevel models examined within‐ and between‐person variability in stress levels, with predictors including caregiver characteristics (age, gender), care recipient functional status (number of activities of daily living requiring assistance), and daily caregiver‐perceived social isolation.

**Results:**

Care‐resistant behaviors (CRBs) were associated with the highest mean daily stress levels (M=1.81, SD=1.41). Higher stress related to CRBs was significantly predicted by greater care recipient functional limitations (β=0.12, *p* = .009) and caregiver‐perceived social isolation (β=0.05, *p* < .001). Stress related to physical aggression was associated with the largest increases in stress levels over time. Higher stress related to physical aggression was also significantly associated with caregiver‐perceived social isolation (β=0.07, *p* = .001) and the interaction between functional limitations and social isolation (β=‐0.03, *p* = .004). Stress related to property destruction demonstrated the second‐largest increases above baseline stress levels and was also significantly associated with caregiver‐perceived social isolation (β=0.07, *p* < .001) and the interaction between functional limitations and social isolation (β=‐0.02, *p* = .042).

**Conclusion:**

Daily stress related to care‐resistant behaviors, physical aggression, and property destruction is significantly worsened by H&L caregiver‐perceived social isolation and care recipient functional limitations, with higher stress reported when isolation is high and functional limitations are lower. These findings underscore the need for culturally responsive interventions that address social isolation and equip H&L caregivers to manage challenging BSDs, ultimately reducing stress, improving caregiver well‐being, and enhancing care for people living with ADRD.

